# A high-throughput peptidomic strategy to decipher the molecular diversity of cyclic cysteine-rich peptides

**DOI:** 10.1038/srep23005

**Published:** 2016-03-11

**Authors:** Aida Serra, Xinya Hemu, Giang K. T. Nguyen, Ngan T. K. Nguyen, Siu Kwan Sze, James P. Tam

**Affiliations:** 1 School of Biological Sciences, Nanyang Technological University, 60 Nanyang Drive, 637551, Singapore

## Abstract

Cyclotides are plant cyclic cysteine-rich peptides (CRPs). The cyclic nature is reported to be gene-determined with a precursor containing a cyclization-competent domain which contains an essential C-terminal Asn/Asp (Asx) processing signal recognized by a cyclase. Linear forms of cyclotides are rare and are likely uncyclizable because they lack this essential C-terminal Asx signal (uncyclotide). Here we show that in the cyclotide-producing plant *Clitoria ternatea*, both cyclic and acyclic products, collectively named cliotides, can be bioprocessed from the same cyclization-competent precursor. Using an improved peptidomic strategy coupled with the novel Asx-specific endopeptidase butelase 2 to linearize cliotides at a biosynthetic ligation site for transcriptomic analysis, we characterized 272 cliotides derived from 38 genes. Several types of post-translational modifications of the processed cyclotides were observed, including deamidation, oxidation, hydroxylation, dehydration, glycosylation, methylation, and truncation. Taken together, our results suggest that cyclotide biosynthesis involves ‘fuzzy’ processing of precursors into both cyclic and linear forms as well as post-translational modifications to achieve molecular diversity, which is a commonly found trait of natural product biosynthesis.

Molecular diversity of small-molecule metabolites and ribosomally-synthesized and post-translationally modified peptides (RiPPs) is a hallmark of natural products that is tightly linked to environmental adaptation and host-defense mechanisms^1^,^2^,^3^. Whereas molecular diversity of metabolites such as gibberellins and terpenes, as well as microbe-derived RiPPs such as cyanobactins, lanthipeptides, and azol(in)e-containing peptides are well studied^4^,^5^,^6^, plant-derived RiPPs such as cysteine-rich peptides (CRP) remain under-explored.

Cyclotides are plant CRPs that exhibit a wide range of pharmacological properties^7^,^8^,^9^,^10^. These peptides contain a cystine-knot and a head-to-tail cyclic backbone^11^, which both contribute to the exceptional stability of cyclotides against degradation by heat, chemicals, and enzymes^12^. Although linear forms of cyclotides do occur in plants, they are rare^13^,^14^. Only 17 of the 282 cyclotides collected in the CyBase database are known linear sequences. Previous studies suggested that the biosynthesis of both linear and cyclic cyclotides is determined at the genetic level through the presence or absence of crucial bioprocessing signals^15^,^16^. The core domain of cyclotide precursors can be processed into either a cyclic or linear form, but not both. The cyclotide domain is a cyclization-competent domain containing an essential C-terminal Asx residue, which serves as the recognition signal for asparaginyl endopeptidase (AEP) to mediate macrocyclization during cyclotide bioprocessing. Notably, the absence of this essential Asx residue produces uncyclizable cyclotides (uncyclotide), as are found in *Hedyotis biflora, Chassalia chartacea, Momordica cochinchinensis,* and *Panicum laxum*^17^,^18^,^19^,^20^.

Recently we successfully isolated the bioprocessing enzyme cyclase butelase 1, together with five transcript homologs, butelase 2–6, from the leguminous plant *Clitoria ternatea*^21^. The presence of multiple butelases with ligase or protease functions suggests that the bioprocessing of cyclotide precursors could be more complex than previously thought.

To unravel the underlying mechanisms of cyclotide diversity and bioprocessing, an efficient and systematic sequencing analysis is both a necessity and a challenge. Because of the extensive sequence homology shown by plant cyclotides, a major challenge is achieving complete sequence coverage to ensure accurate identification and characterization of these peptides. Another challenge is the requirement of a site-specific enzyme that opens the cyclotide ring to prevent random cleavage of the cyclic peptide backbone that could produce inaccurate sequences. Poth *et al.* previously isolated 12 *C. ternatea* cyclotides CterA-L, which contain five pairs of sequences that differ only at the Asn/Asp ligation site^22^. Furthermore, we independently reported another panel of 12 novel *C. ternatea* cyclotides (cT1-12) that includes both cyclic and acyclic products forms, which we collectively refer to as cliotides. These cliotide peptides can be bioprocessed from the same cyclization-competent precursor^23^.

Here we describe an improved peptidomic method coupled with a transcriptomic approach to study cyclotide diversity and show that the cyclotide domain of cliotide precursors allows the production of both cyclic and linear cliotides through alternative bioprocessing of their precursors and by side chain post-translational modifications (PTMs). We also used the novel Asx-specific endoprotease butelase 2 to overcome the requirement of a site-specific ring-opening enzyme, which allowed the direct use of RNA transcriptome sequences as the database. In this work, transcriptome analysis revealed more than 50 cliotides and peptidomic analysis discovered hundreds more cliotide derivatives. The availability of the processing enzymes(s) together with the abundance of cliotide sequences found in the *C. ternatea* transcriptome provide a promising model to study cyclotide molecular diversity in plant CRPs.

## Results

### Strategy framework

**Figure 1:**
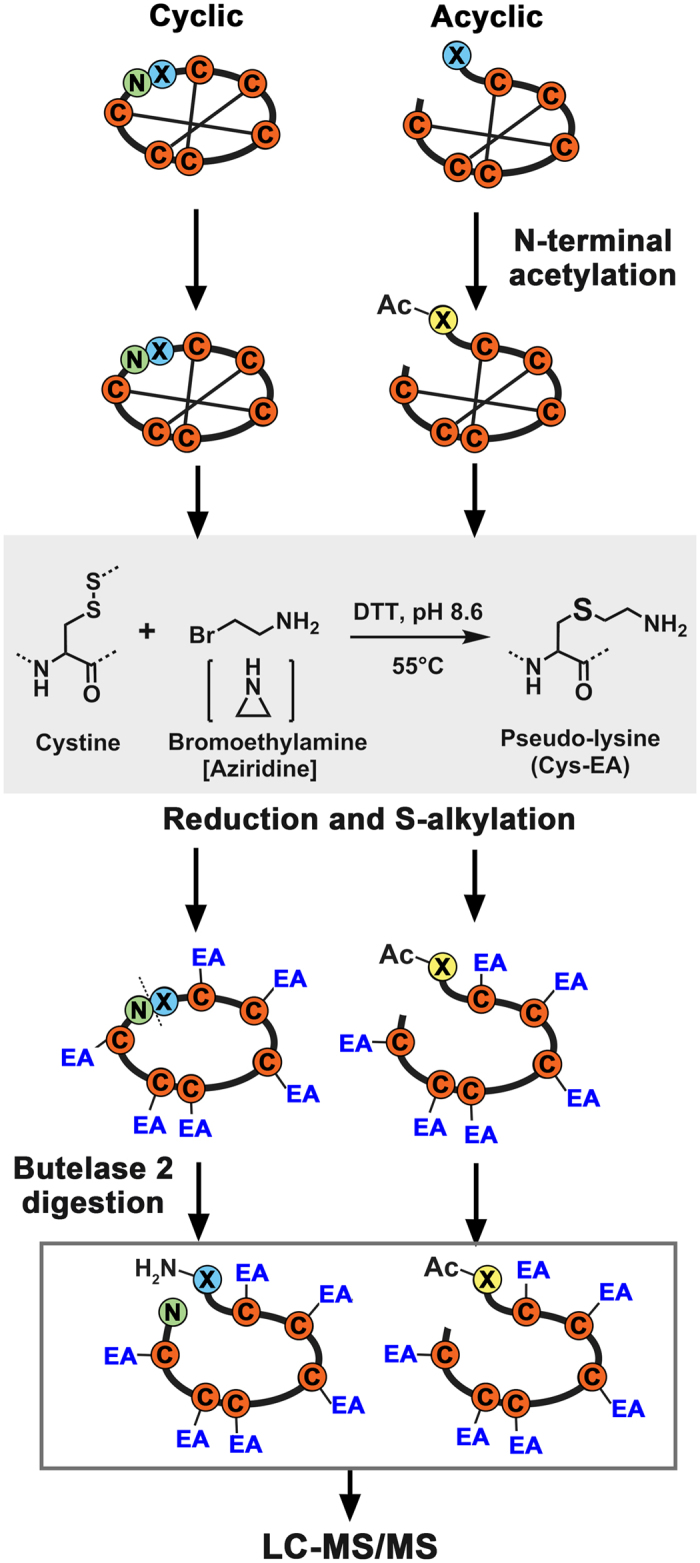
Flowchart of sample preparation for LC-MS/MS analysis of cliotides. X: N-terminal residue in the cliotide sequences is usually Gly and occasionally Asp or Ser. Ac: acetylated N-terminus. EA: ethylamine-alkylation.

Our approach employed both peptidomic and transcriptomic methods to analyze mixtures of expressed cliotides in plant extracts (Fig. 1). These methods included: (1) chemical derivatization by N-terminal acetylation to differentiate naturally occurring acyclic forms from cyclic forms; (2) disulfide bond reduction and conversion of the resultant Cys residues into basic pseudo-Lys (ψLys)^24^,^25^; (3) enzymatic linearization of the ψLys-cliotide by the novel AEP butelase 2 that targets the biosynthetic Asx-Xaa ligation site to produce a linearized ψLys-cliotide identical to its precursor sequence; (4) LC-MS/MS analysis of the resulting highly-charged and linearized ψLys-cliotide by electron transfer dissociation (ETD)^26^; and (5) construction of a cliotide database from the reported sequences and contigs extracted from the transcriptome, and database searches to identify cyclic and linear cliotides as well as their modified derivatives.

### Extraction and linear peptide N-terminal acetylation

**Figure 2:**
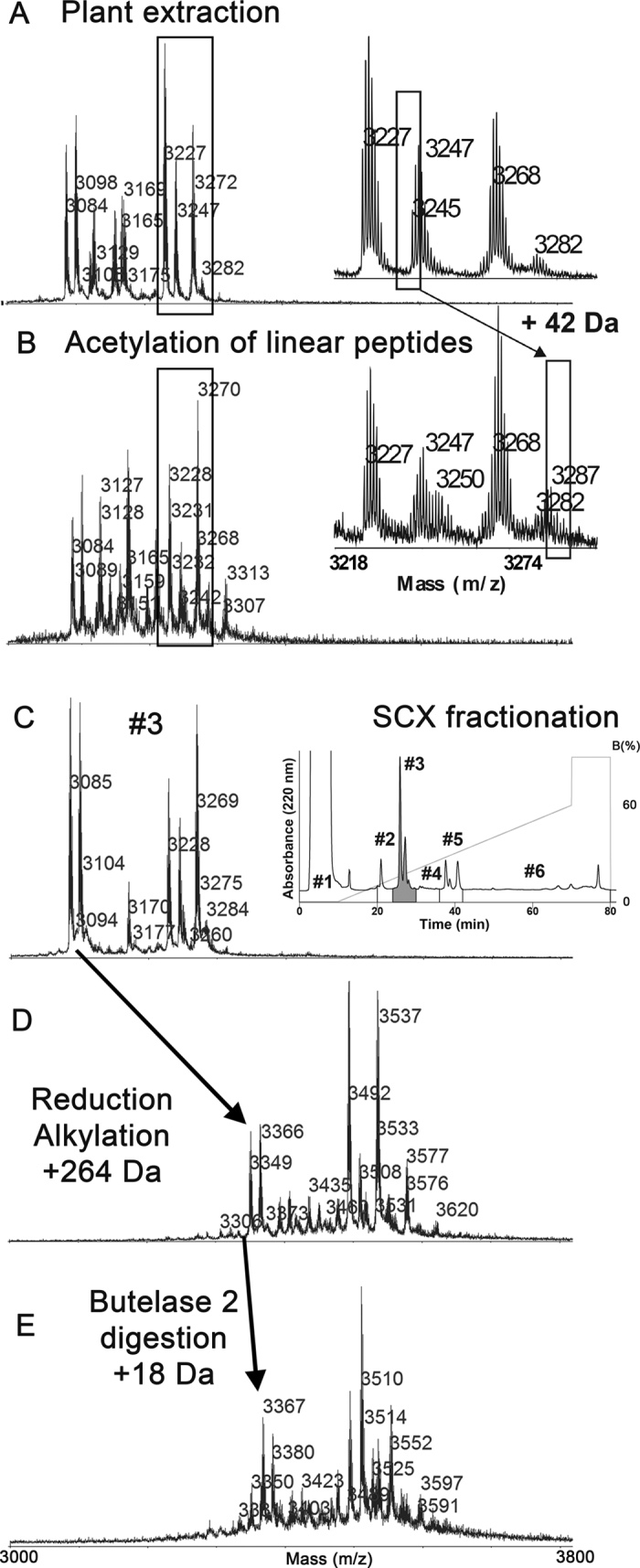
MS characterization of each chemical derivatization process. (**A**) MS profile of the crude *C. ternatea* extraction. (**B**) Acetylation of free N-terminal amine groups of the linear peptides resulted in a mass increase of 42 Da. (**C**) Fractionation of the crude mixture by strong cation exchange chromatography (example showed MALDI-TOF spectrum of fraction #3). (**D**) Disulfide bonds were reduced and alkylated in a one-pot reaction using a DTT and BrEA mixture at pH 8.6, 55 °C. A mass increase of 264 Da was observed after reduction and alkylation of CRPs with 3 disulfide bonds. (**E**) For macrocyclic of CRPs containing one Asn residue, butelase 2-catalyzed digestion resulted in a mass increase of 18 Da.

Cliotides extracted from *C. ternatea* with pre-chilled ethanol/water (4/6, v/v) gave a complex mass spectrometry (MS) profile that included numerous peaks in the 3–4 kDa range (Fig. 2A). The crude extract was slowly acidified to pH 2 to denature and precipitate proteins, which were then removed by filtration. This raw extract of cliotides contained approximately 1 mg peptides per gram of fresh *C. ternatea* plant material.

We capped the free N^α^-amine of the naturally occurring linear peptides by acetylation^27^ to allow them to be distinguished from the linearized cyclic counterparts generated by butelase 2 digestion in the subsequent step. The acetylation reaction was conducted for only 5 min to avoid undesired side-chain acetylation that could complicate PTM identification. Thus, cliotides with low abundance likely escaped acetylation. In the MS profiles, acetylation induced a mass increase of 42 Da (Fig. 2B). After acetylation, the cliotide mixture was fractionated on a strong cation-exchange column (Fig. 2C). Fractions with low peptide content were concentrated to improve detection of low-abundance cliotides.

### Conversion of Cys to ψLys by one-pot reduction and alkylation

We next treated cliotide samples with dithiothreitol (DTT) and 2-bromoethylamine (BrEA) using a one-pot approach to convert Cys into ψLys. Our conditions allowed multiple reactions to occur simultaneously: (1) DTT reduction of disulfide bonds; (2) cyclization of BrEA into its reactive aziridine (ethylene imine) form; and (3) aziridine-ring opening by cysteinyl thiol nucleophiles, resulting in S-alkylation by aziridine to complete the conversion of cystines into two ψLys as Cys-S-ethylamine (S-EA).

We tested a series of conditions using cliotide cT4 as a model to optimize the one-pot reaction. The optimal conditions for treating cliotide mixtures (0.1–1 mM) that we identified were: 30 mM DTT and 60 mM BrEA in 0.2 M Tris-HCl (pH 8.6) at 55 °C. Under these conditions the temperature favored rapid cyclization of BrEA into aziridine and the pH was slightly higher than Cys-thiol pKa but lower than that of other nucleophiles, which minimized side reactions such as non-specific aziridine alkylation and polymerization. Conversion of cystine to ψLys was achieved in 1 h, resulting in a mass increase of 264 Da for the six Cys in cliotides as monitored by MS (Fig. 2D). In comparison, a conventional two-step procedure to reduce and alkylate CRPs would involve a reduction reaction for 1 h and a subsequent alkylation step for at least 3 h (Supplementary Figure 1).

### Butelase 2-mediated cliotide linearization

The butelase 2 precursor sequence used was based on a previously reported transcriptome analysis^21^. To obtain recombinant butelase 2 to catalyze ring-opening of the cyclotide backbone at the conserved C-terminal asparagine ligation site, we used a baculovirus expression system to express butelase 2 precursor in Sf9 cells^28^, which yielded ~1 mg soluble butelase 2 precursor per liter of Sf9 cell culture.

Similar to other known AEPs, the butelase 2 precursor underwent auto-activation under acidic conditions (pH 5.0). After expression and auto-activation, the 52 kDa butelase 2 precursor was trimmed at Asp57 and Asn394 (predicted) into the 38 kDa active form (Supplementary Figure 2). Activated butelase 2 enzyme mediated site-specific ring-opening of the cliotide backbone at the Asx-Xaa ligation site within 30 min at 37 °C to yield a mass shift of +18 Da (Fig. 2E). Notably, butelase 2 was active only against cliotides with reduced and S-alkylated disulfides, and showed no activity towards cliotides that had intact cystine-knot structures.

### LC-MS/MS analysis of derivatized and linearized cliotide samples

**Figure 3:**
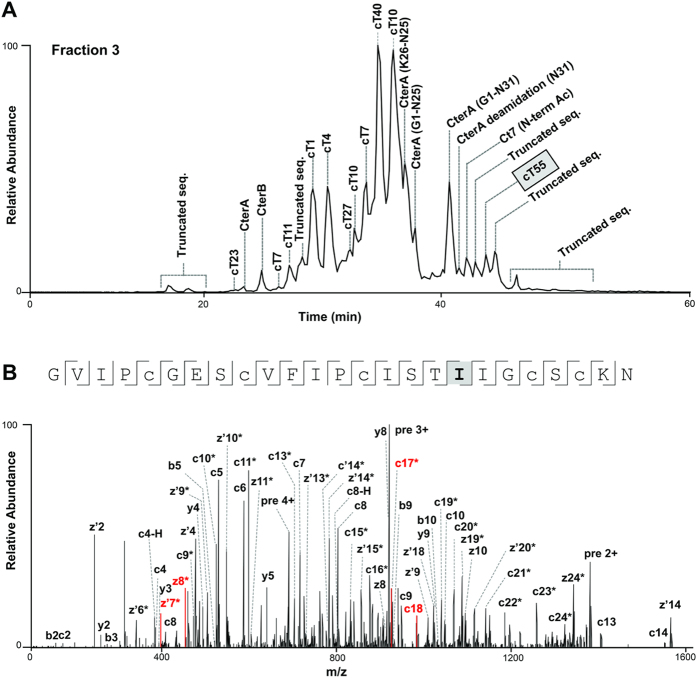
LC-MS/MS profile of CRPs in the fractionated extract of *C. ternatea*. (**A**) Example LC-MS profile of SCX fraction 3. Reduced and alkylated species are labeled in each identified peak and the newly identified variant cT55 is framed. (**B**) Annotated MS/MS spectra of the new cT55 sequence which exhibited a single amino acid substitution (V to I) compared with CterA. *Double-charged fragment. z’ ions correspond to z + 1 ions.

After acetylation, ψLys conversion, and butelase 2-mediated site-specific digestion, cliotide samples displayed an average molecular mass of ~3500 Da and a charge state of +5 to +7. The peptides were then subjected to sequencing by ETD. In order to record high quality MS/MS spectra, trial runs were performed to optimize performance by varying the ETD activation time, ion spray voltage, automatic gain control values, and the number of microscans. The optimized method included three MS/MS scans per precursor using three different ETD activation times that ranged from 65–95 ms to ensure sufficient peptide backbone fragmentation to cover the entire sequence diversity of the cliotide samples. On-line LC-separation of different cliotides and isoforms in the complex mixture improved identification of low-abundance cliotides (Fig. 3A).

### RNA transcriptome and database construction

Total RNA extraction was performed using fresh *C. ternatea* root samples and the transcriptome was sequenced using Illumina Hiseq 2000 to yield 65 million clean reads with 98.5% Q20 percentage and 44.6% GC percentage (SRR1613316). After assembly by Trinity^29^ (Supplementary Table S1), 137,334 contigs were obtained that had an average length of 352 nucleotides. Using in-house custom software Protein Analyzer 1.6, 52 contigs containing cliotide-encoding domains (C-C-C-CXC-C) were exported. After including the 10 sequences described by Poth *et al.*^22^, two additional sequences from CyBase, and three unpublished sequences determined at the protein level by our laboratory that were not found in the root transcriptome, the cT-database (CT_CRP_ORF) eventually consisted of 67 sequences, including cT1–53 and CterA-O (nomenclature of published cliotides remained unchanged as provided in Supplementary Data Set 1). Analysis of cT_CRP_ORF revealed that the Asx of all cyclotide-encoding domains is followed by two highly conserved residues (HV, HI or VV) to form the recognition tripeptide for butelase 1 (Supplementary Figure 3). We also constructed a modified database (CT_CRP_MOD) using the duplicated encoding region to analyze linearized cliotides that are digested at an internal Asx instead of the C-terminal Asx.

### Data analysis and product diversity

The database search and PTM analysis were assessed in PEAKS studio using PEAKS PTM function^30^ to search our data for >650 PTMs listed in the Unimod database^31^ and using SPIDER algorithm to identify single mutations. We identified 272 cliotides by LC-MS/MS analysis and confirmed 38 cliotide contigs (Table 1) that represented 55% coverage of the total number of cliotide genes present in the *C. ternatea* transcriptome (Supplementary Data Set 2). Our data set contained 47 full sequences, including all 12 cliotides previously reported by our group, and seven that were reported by the Poth *et al.* Additionally, 30 truncated sequences with an intact cystine-knot (i.e. six Cys residues) and 195 truncated sequences with five or fewer Cys residues are reported here.

Five cyclic-competent cliotides, cT6, cT7, cT10, cT19, and CterA, were found to produce both cyclic and linear full sequences. They were distinguished by the prior N-terminal acetylation procedure. In addition, seven truncated peptides with N-terminal acetylation were identified for cliotides cT7, cT10, cT17, and cTerA.

Truncated linear sequences containing six Cys residues were identified from cliotides cT2, cT10, cT17, cT32, CterA, and CterI/J. Shorter truncated cliotides with five or fewer Cys residues occurred in low abundance. The detected numbers of MS/MS spectra per run were typically <10, whereas the spectral count of cyclic counterparts was uniformly >100. In order to confirm that the truncated linear sequences identified in our experiment were not generated during sample processing, the presence of truncated linear sequences was also investigated in untreated raw extracts by performing a LC-MS analysis. This analysis detected a wide range of low-abundance short peptides (<3000 Da) and thus confirmed their presence in plants (examples provided in Supplementary Figure 4).

In total, 33 cliotides with side chain modifications were identified, including deamidation (13 at Asn and three at Gln), oxidation (four at Met and one at Trp), hydroxylation (two at Pro), dehydration (one at Ser, two at Asp and one at Thr), mono-hexosylation (five at Ser), and methylation (one at Thr) (MS/MS spectra of cliotides with intact cystine-knot and PTMs are summarized in Supplementary Figure 5).

Deamidation could be an asparaginase-mediated modification^32^ or a non-enzymatic aging process that is directly dependent on pH and temperature^33^,^34^,^35^,^36^. We performed parallel experiments by incubating peptides at 55 °C for 1 h at different pH values (2, 6, and 8.6) to rule out the possibility of deamidation due to sample processing, and found that the same number of deamidation sites was detected at all three pH conditions.

Oxidation of Met to Met(O) (sulfoxide) and Trp to hydroxy-Trp were previously reported for cyclotides^37^. Met oxidation could occur spontaneously during processing and we found the amount of oxidized Met(O)-containing cliotide cT3 gradually increased during storage.

Hydroxylation is an enzyme-catalyzed process, and we found that the amount of P13-hydroxylated Pro-containing cliotide cT6 (Supplementary Figure 6A) remained unchanged even after prolonged storage of the clarified extract at room temperature.

Dehydration of Asp led to the formation of succinimide (−18 Da)^38^ and the aspartimide intermediate was confirmed by LC-MS/MS analysis (Supplementary Figure 6B). Spontaneous opening of aspartimide (+18 Da) resulted in normal or iso-peptides. Meanwhile, dehydration at Ser/Thr could be due to thermal or alkaline-induced β-elimination of Ser/Thr or glycoSer/Thr that results in the formation of dehydroalanine or dehydrobutyric acid.

Glycosylation with a single hexose at conserved Ser residues in loop 1 or loop 4 of cliotides cT6 (Supplementary Figure 6C), cT7, and cT10, and methylation at T17 in CterA (Supplementary Figure 6D) were also observed.

We observed extensive modifications in abundant cliotides, including cliotide cT3, cT6, cT7, cT10, and CterA. For example, the cliotide gene ctc7 coding for cT7 gave 28 products, which included 25 truncated sequences and four products with Asn-deamidation or Ser-hexosylation (Table 2). In contrast, as expected, only one or two products with limited modifications were found for the low-abundance cliotides such as cT21, cT34, cT43, and CterD.

We identified two new cliotides that were not found in the *C. ternatea* transcriptome. Cliotide cT54 differed from cT10 only at residue 25 (N25 and D25, respectively) and was cleaved by butelase 2 to give a truncated sequence (Supplementary Figure 6E). Meanwhile, cliotide cT55 differed from CterA at residue 18 (I18 and V18, respectively), and was validated and annotated manually (Fig. 3B). Both cliotides are unlikely to be derived from post-translational modifications or misidentification.

## Discussion

The finding that alternative bioprocessing mechanisms of a single cyclotide-encoding domain generate both cyclic and acyclic products with an identical amino acid composition expands the current knowledge that an Asx-containing cyclotide precursor is processed exclusively to a cyclotide. A plausible explanation for this alternate outcome is that acyclotides are produced as minor products that may escape detection. In our previous work on *in vitro* cyclization of the cyclotide precursor kalata B1-HV, we observed that butelase 1 produced not only macrocyclic kB1 as the major product but also a small quantity of linear kB1 that did not undergo the butelase-mediated ligation reaction^21^. The substrate specificity of butelase 1 exhibits a broad tolerance of an acceptor N-terminal residue at the P1” position, but a narrow tolerance at the P2” position, where hydrophobic amino acids are preferred. Thus, the flanking sequences at the essential Asx recognition site appear to influence the bioprocessing of cyclotide precursors to produce various ratios of cyclic and acyclic forms. Mylne *et al.*^19^ reported the discovery of the cyclic knottin gene TIPTOP that encodes both cyclotide-coded sequences and an acyclotide-coded sequence as a tandem array. The mature acyclotide domain at the C-terminus of the TIPTOP gene does not encode the Asx processing signal. Although both cyclic and acyclic knottins are produced by the same gene through this “one-gene-multiple-products” mechanism, they do not arise from the same mature domain. As such, the Mylne *et al.* study supports the current dogma that each mature domain can be bioprocessed to give either a cyclotide or acyclotide, but not both. However, the structure of cliotide-encoding genes differs from that of TIPTOP. Our work suggests that bioprocessing of a cyclization-competent cyclotide domain is ‘fuzzy’, thus both cyclic and acyclic forms can occur.

Once linear peptides are formed, they are susceptible to degradation by endo- and exo-proteases to generate truncated acyclotides^39^, such as the four full-length linear cT2 and 10 partial sequences that were identified in our study. The natural existence of these truncated forms in raw *C. ternatea* extracts was investigated by MS analysis that showed the existence of multiple truncated peptides with mass <3000, which verified that these linear peptides are not artifacts of sample processing (Supplementary Figure 4). Truncation of peptide sequences is also a means for diversification of neurotoxic CRPs in animal venoms, wherein >100 different truncated conotoxins can be generated from a single gene, MrIA^5^,^40^.

**Figure 4:**
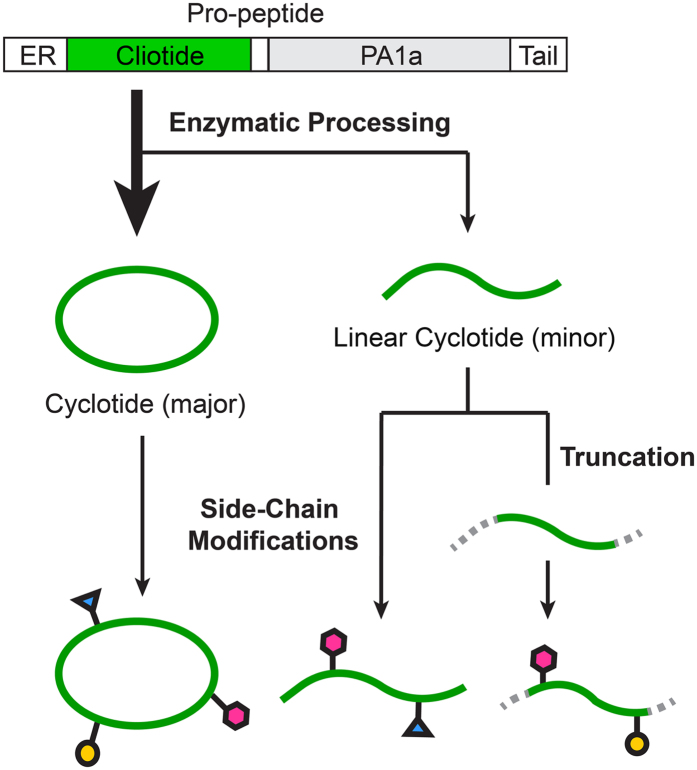
Biosynthesis and diversification mechanisms of cliotides through fuzzy processing and post-translational modifications.

In addition to cliotide diversity that is derived from structural forms and truncation products, PTMs on peptide amino acid side chains further increase their molecular diversity. We showed that more than 10% of the products analyzed (33 of 272) underwent side chain modifications, and that, on average, one cliotide gene could produce seven different products at the protein level (Fig. 4).

Asn-deamidation and Met/Trp oxidation were major side chain modifications in both this study and a previous study on cyclotides that examined four purified HPLC fractions of *Oldenlandia affinis*^41^. Here we found that Asn-deamidation is frequently observed when the susceptible Asn residue is adjacent to a non-hindered Gly (8 out of 13), which is consistent with previous findings^35^,^42^,^43^. In addition, Asn-deamidation adjacent to Ser (1 out of 13) and Lys (4 out of 13) was also observed. Asn in cliotides may not favor spontaneous deamidation due to the steric hindrance in their stabilized macrocyclic structure as was demonstrated for tubular proteins^44^. We also verified Asn-deamidation sites at different pH by excluding spontaneous deamidation during the sample processing step, an observation that agreed with those of Chelius *et al.*^45^.

Similar to deamidation, oxidation of Met could be a spontaneous reaction that occurs when peptides were exposed to an oxidative environment. In contrast, oxidation of Trp, which generally takes place in plant mitochondria, is likely an aging process^46^,^47^.

To the best of our knowledge, hydroxyproline (+16 Da), mono-hexosylation (+162 Da), dehydration (−18 Da) and O-methylation (+14 Da) have not been reported in plant CRPs. Hydroxylation is an enzymatic reaction that is mediated by hydroxylase. Our data showing that one truncated sequence from cT17 is hydroxylated at P13, which is in agreement with earlier studies showing that peptide hormones show proline hydroxylation^48^. In plants, hydroxylation of peptides is catalyzed by 4-hydroxylase (P4H), which acts only on Pro residues at the 4-position carbon^49^. Plant P4H is known to localize mainly to the Golgi and in part to the ER^50^,^51^. The *C. ternatea* transcriptome carries at least six P4Hs, and these enzymes are homologous to the *Arabidopsis thaliana* prolyl 4-hydroxylase At-P4H1^52^ (Supplementary data set 3).

Side chain modifications involving oxidation of Met and Trp or hydroxylation of Pro, Tyr, and Thr reduce surface hydrophobicity and are a common feature of peptide toxins^53^. Dehydration and methylation are frequently seen for secondary metabolites, but their occurrence and functions in plant CRPs have not been reported. We also found three cliotides with Ser O-hexosylation, while the mechanism of hexosylation and biological function of cliotides remain unclear. We speculated that processing enzymes of secondary metabolites and proteins affect CRP maturation in plants as a means to further increase diversity.

Opening the macrocyclic backbone and intramolecular disulfides are necessary for accurate sequencing of cyclotides by MS. Previously, Colgrave *et al.* used endopeptidase Glu-C to linearize cyclotides at a conserved Glu residue^41^. The resulting linearized peptides differed from their source gene sequences, and the self-built program ERA was used to construct a modified database of proteolytic cyclotide sequences. Here we used the novel AEP, butelase 2, to cleave peptides at the natural Asn ligation site to yield linearized peptides that match the cliotide-encoding domain in the precursor sequence. Consequently, we could use the RNA transcriptome as a database for our searches without the need for further modifications.

ETD fragmentation was previously shown to generate extensive primary structure information on peptides and proteins that exhibit a high charge state (>+3) and mass >1500 Da^54^. These studies indicated that electron-mediated cleavage can induce extensive peptide backbone fragmentation, while labile PTMs remained intact^55^,^56^. Although conversion of Cys into ψLys by alkylation with aziridine^57^,^58^ is a useful method that decreases the m/z ratio and in turn improves ETD fragmentation, existing methods are hampered by side reactions under harsh reaction conditions or prolonged reaction time. To avoid these limitations, we assessed a different one-pot reaction condition to optimize the reduction and alkylation reactions, wherein DTT functioned both as a potent reductant and as a nucleophile to minimize side reactions such as non-specific aziridine alkylation and polymerization.

Why do plants process a panel of cyclic and linear products from a single precursor? Although we do not fully understand the physiological roles of these peptides in plants, producing both cyclic and linear forms of cyclotides would increase structural diversity to confer adaptive advantages, and natural product biosynthetic pathways that generate further modifications have evolved to favor molecular diversity. Firn and Jones in 2003 proposed the ‘screening model’ to explain molecular diversity^59^. In their view, “evolution would favor organisms that could generate and retain chemical diversity at low cost”. They further proposed that the making and ‘screening’ of a large number of chemicals by organisms would in turn enhance fitness because the greater the chemical diversity, the greater the chances of producing the rare chemical that has useful, potent biological activity^59^. This ‘diversity-based’ model emphasizes the nature of the biosynthetic pathways rather than the way in which their products are used. Thus, our work demonstrating that the bioprocessing of a cyclotide domain can produce different molecular forms of cyclotides provides support for the ‘diversity-based’ model.

In conclusion, we developed an improved peptidomic strategy for systematic sequencing and characterization of cliotides using partially purified plant extracts. Our strategy was enabled, in part, by the novel Asx-endopeptidase butelase 2 that we used to linearize cliotides at their biosynthetic ligation site for use in direct transcriptomic analysis. The omics analysis revealed that cyclotide precursors exist in two forms in nature, cyclization-competent and incompetent, and the ultimate structure depends on the presence (or absence) of the C-terminal Asx processing signal. Although genetically cyclization-competent, this type of precursor can produce a panel of cyclic and products through ‘fuzzy’ processing. First, the linear forms would be susceptible to degradation by exoproteases as truncation products. Second, side chain modifications of both cyclic and linear peptide products lead to further diversification. Taken together, these results suggest that plant cyclotides, a family of RiPPs, achieve molecular diversity in their biosynthesis by employing a low cost “one precursor-multiple products” strategy, which is a trait that is common to small-molecule metabolites and microbe-derived RiPPs for adaptation and defense. Future characterization of biosynthetically modified cyclotides could uncover compounds with that have interesting therapeutic potential.

## Methods

### Plant extraction and purification

A *C. ternatea* plant (weight 28.9 g) collected from a local nursery was weighted and used for our study. The ethanol concentration in the extraction buffer was optimized based on the MS profile showing the highest abundance of peaks in the range of 3–4kDa. Plant was blended with chilled ethanol/water (4/6, v/v) (10 mL/g of sample) and centrifuged at 4000 rcf for 30 min at 4 °C. Supernatant was diluted to 20% in ethanol and then acidified to pH 2 by drop-wise addition of 1 N HCl. The mixture was then filtered through a 0.45 μm filter to get the raw extract and purified in a self-packed C-18 (20 g resin) flash chromatography column by washing with 20% ethanol containing 10 mM HCl (4 L) and eluting with 200 mL 80% acetonitrile (ACN) containing 0.1% trifluoroacetic acid (TFA). The clarified extract was lyophilized to dried powder and then re-dissolved in 10% ACN containing 0.1% TFA to a final concentration of 3.5 mg/mL. The re-dissolved peptide solution was again passed through a 0.45 μm filter before further processing.

### RNA extraction, sequencing and assembly

Total RNA from fresh *C. ternatea* root was extracted using Trizol reagent (Life Technologies, Waldbroon, Germany) and sent to Beijing Genomic Institute (BGI, China) for sequencing and assembly. Quality check was done using Agilent 2100 Bioanalyzer (Agilent Technologies). Poly(A) RNA was enriched using oligo(dT) magnetic beads and fragmented as template for cDNA synthesis. Short fragments were purified and resolved with EB buffer (Qiagen, Hilden, Germany) for end reparation and single nucleotide A (adenine) addition. Suitable fragments were selected for PCR amplification. Finally, the library was sequenced using Illumina HiSeq 2000. Raw data were analyzed and assembled using Trinity (Supplementary Table S1).

### Labeling of linear peptides by N-terminal acetylation

Peptide solution was pre-chilled on ice and adjusted to pH 3.0–3.3 using ammonium acetate buffer (0.2 M, pH 5). Acetic anhydride solution (10%, 1 M in ACN) was added to the peptide solution which was then incubated on ice for 5 min before being lyophilized. The peptide powder was re-dissolved in buffer SCX-A (20 mM Na_2_HPO_4_, 20% ACN, pH 2.9).

### HPLC fractionation

Sample fractionation using strong cation-exchange liquid chromatography (SCX-LC) with a PolySULFOETHYL A column (PolyLC, 200 × 4.6 mm) was conducted with a gradient of 10–60% buffer SCX-B (1 M KCl, 20 mM Na_2_HPO_4_, 20% ACN, pH 2.9). Five fractions were collected as shown in Fig. 2C. For the MS analysis of the raw extract, an aliquot of the non-treated raw extract was fractionated by SCX-LC under the same conditions. Fractionated peptide mixtures were desalted using a C-18 Sep-pack column (50 mg, Waters) and lyophilized.

### One-pot reduction and alkylation

Lyophilized peptides in each fraction were re-dissolved in variable volume of H_2_O to a final concentration of 3.5 mg/mL. The reaction mixture comprising about 0.5 mM peptides, considering molecular weight of *C. ternatea*-derived CRPs from 3000–3500 kDa, 30 mM DTT and 60 mM bromoethylamine (BrEA) in 0.2 M Tris-HCl buffer (pH 8.6) was incubated at 55 °C for 60 min and quenched by adjusting to pH 6 using 3 N HCl. The reduced, alkylated peptide samples were desalted using a C-18 Sep-pack column and dried by SpeedVac (no heating). After re-dissolving in H_2_O, peptide solutions were stored at −20 °C.

### Recombinant expression of butelase 2

Sequence of butelase 2 has been reported in the previous paper by Nguyen *et al.*^21^. The amino acid sequence of butelase 2 without the predicted signal sequence (Supplementary Figure 2B) was inserted into the pFB-LIC-Bse expression vector after the His6-TEV tag (MGHHHHHHSSGVDLGTENLYFQS) (Supplementary data set 4). The constructed plasmid was transfected into sf9 cells^28^ and the expressed protein was purified by Nickel NTA column (Life Technology). The purified enzyme was treated with TEV protease to remove the His6-TEV tag and concentrated by a centrifugal unit with a 10 kDa molecular-weight cut-off. Subsequently, the enzyme was incubated at room temperature for 2 h in 50 mM sodium acetate buffer (pH 5.0) for auto-activation. The activity of butelase 2 was assayed by mixing 0.1 μM enzyme with 50 μM Z-AAN-AMC in sodium phosphate buffer (pH 6.0) and incubated at 37 °C for 30 min. The relative fluorescence intensity at 460 nm increased by about 10 folds, which confirmed that the recombinant butelase 2 was functionally active.

### Butelase 2-mediated cyclic CRP digestion

Butelase 2 was auto-activated by acidification to pH 5 using 10 mM sodium phosphate buffer and incubated at 37 °C for 2 h before use in the digestion reaction. The digestion solution containing 2 μM butelase 2, 0.5 mM reduced and alkylated peptide mixture, 100 mM sodium phosphate buffer (pH 6.5), and 5 mM EDTA was incubated at 42 °C for 30 min, after which the reaction was quenched by acidification to pH 4 using 1 N HCl.

### MALDI-TOF mass spectrometry

Sample purification, acetylation, reduction, alkylation and digestion reactions were monitored by MALDI-TOF mass spectrometry using an ABI 4800 apparatus. Reflectron acquisition mode was used in the 1000–6000 Da mass range with focusing mass of 3000 Da. A saturated MALDI matrix solution was prepared by dissolving α-cyano-4-hydroxycinnamic acid (CHCA) in 75% ACN with 0.1% TFA. C-18 zip-tip was used to desalt the reaction mixtures before spotting. Each MALDI spot contained 0.5 μL desalted peptide solution and 0.5 μL matrix solution.

#### LC-MS/MS spectrometry

The LC-MS/MS analyses of peptides were performed using an Orbitrap Elite mass spectrometer (Thermo Scientific Inc., Bremen, Germany) coupled with a Dionex UltiMate 3000 UHPLC system (Thermo Scientific Inc., Bremen, Germany). Samples were sprayed using a Michrom’s Thermo CaptiveSpray nanoelectrospray ion source (Bruker-Michrom Inc, Auburn, USA) and the separation was conducted using a reverse phase Acclaim PepMap RSL column (75 μm ID × 15 cm, 2 μm particles, Thermo Scientific). The mobile phase was 0.1% formic acid (FA) as eluent A and 90% ACN 0.1% FA as eluent B, with a flow rate of 0.3 μL/min. A 60 min gradient was used for the elution as follows: 3% B for 1 min, 3–35% B over 47 min, 35–50% B over 4 min, 50–80% B over 6 s, 80% for 78 s and then reverted to the initial state over 6s and maintained for 6.5 min.

The Thermo Scientific Orbitrap Elite mass spectrometer was set to positive ion mode using LTQ Tune Plus software (Thermo Scientific Inc., Bremen, Germany) for data acquisition, alternating between a Full FT-MS (350–3000 m/z, resolution 60.000, with 1 μscan per spectrum) and a FT-MS/MS scan applying 65, 80 and 95 ms ETD activation times, (150–2000 m/z, resolution 30.000, with 2 μscan averaged per MS/MS spectrum). The 3 most intense precursors with charge >2+ were isolated with a 2 Da mass isolation window and fragmented. The automatic gain control (AGC) for Full MS and MS^2^ was set to 1 × 10^6^ and the reagent AGC was 5 × 10^5^.

### LC-MS spectrometry

The MS analysis of the non-treated raw extract fractions was performed using the same instrumentation and conditions described in LC-MS/MS spectrometry section including only Full FT-MS scan.

### Validation of asparagine deamidation

Asn-deamidation was validated by incubation of a reduced and alkylated sample (Fraction 3) for 1 h at variable pH (2, 6 and 8.6) before analysis by mass spectrometry to determine the extent of spontaneous N deamidation during sample incubation at high pH.

### Data analysis

The data analysis were performed using PEAKS studio (version 7.0, Bioinformatics Solutions, Waterloo, Canada) where 10 ppm MS and 0.05 Da MS/MS tolerances were applied. A false discovery rate of 0.1% was applied to accept the sequences. Additionally, PTMs in peptides maintaining intact the 6 Cys residues identified from our experiment were validated manually.

## Additional Information

**How to cite this article**: Serra, A. *et al.* A high-throughput peptidomic strategy to decipher the molecular diversity of cyclic cysteine-rich peptides. *Sci. Rep.* **6**, 23005; doi: 10.1038/srep23005 (2016).

## Supplementary Material

Supplementary Information

Supplementary Dataset 1

Supplementary Dataset 2

Supplementary Dataset 3

Supplementary Dataset 4

## Figures and Tables

**Table 1:** Full sequences of 38 cliotides determined by both transcriptome and proteome.

No.	Contig	Sequence	No. identified
1	cT1	GIP--CGESCVFIPCITGAI-GCSCKSK-VCYRN	14
2	cT2	GEFLKCGESCVQGECYTP---GCSCDWP-ICKKN	14
3	cT3	GLPT CGETCTLGTCYVPD---CSCSWP-ICMKN	18
4	cT4	GIP--CGESCVFIPCITAAI-GCSCKSK-VCYRN	15
5	cT5	GIP--CGESCVFIPCISTVI-GCSCKNK-VCYRN**	2
6	cT6	SIP--CGESCVYIPCLTTIV-GCSCKSN-VCYSN	20
7	cT7	GIP--CGESCVFIPCTVTALLGCSCKDK-VCYKN	31
8	cT8	GIP--CGESCVFIPCISSVV-GCSCKSK-VCYNN	3
9	cT9	GIP--CGESCVFIPCLTTVV-GCSCKNK-VCYNN	6
10	cT10	GIP--CGESCVYIPCTVTALLGCSCKDK-VCYKN	27
11	cT11	GIP--CGESCVFIPCTITALLGCSCKDK-VCYKN	9
12	cT12	GIP--CGESCVFIPCITGAI-GCSCKSK-VCYRD	17
13	cT15	GLPI-CGETCFKTKCYTK---GCSCSYP-VCKRN	12
14	cT17	GTVP-CGESCVFIPCITGIA-GCSCKNK-VCYLN	19
15	cT18	GLPI-CGETCFTGTCYTP---GCTCSYP-VCKKN	2
16	cT19	GSVIKCGESCLLGKCYTP---GCTCSRP-ICKKN	9
17	cT20	GSAIRCGESCLLGKCYTP---GCTCDRP-ICKKN	2
18	cT21	DLQ--CAETCVHSPCIGP----CYCKHGLICYRN	1
19	cT23	GFP--CGESCVFIPCTVTALLGCSCKDK-VCYKN	2
20	cT27	GVIP-CGESCVFIPCITGAI-GCSCKSK-VCYRN	6
21	cT28	GGSIPCGESCVFLPCFLP---GCSCKSS-VCYLN	4
22	cT29	GDPLKCGESCFAGKCYTP---GCTCEYP-ICMNN	2
23	cT32	GDLFKCGETCFGGTCYTP---GCSCDYP-ICKNN	6
24	cT33	GFN-SCSEACVYLPCFSK---GCSCFKRQ-CYKN	3
25	cT34	SYIP-CGESCVYIPCTVTALLGCSCSNK-VCYKN	1
26	cT40	GIP--CGESCVFIPCTITALLGCSCKSK-VCYKN	4
27	cT43	DLI--CSSTCLHTPCKASV---CYCKNA-VCYKN	1
28	cT45	-----CGESCVFLPCFIIPG--CSCKDK-VCYLN	1
29	cT54	GIP--CGESCVYIPCTVTALLGCSCKNK-VCYRN	1
30	cT55	GVIP-CGESCVFIPCISTLI-GCSCKNK-VCYRN	1
31	CterA	GVIP-CGESCVFIPCISTVI-GCSCKNK-VCYRN	20
32	CterB	GVP--CAESCVWIPCTVTALLGCSCKDK-VCYLN	3
33	CterD	GIP--CAESCVWIPCTVTALLGCSCKDK-VCYLN	2
34	CterG	GLP--CGESCVFIPCITTVV-GCSCKNK-VCYNN	8
35	CterH	GLP--CGESCVFIPCITTVV-GCSCKNK-VCYND	7
36	CterI	GTVP-CGESCVFIPCITGIA-GCSCKNK-VCYIN	17
37	CterJ	GTVP-CGESCVFIPCITGIA-GCSCKNK-VCYID	33
38	CterO	GIP--CGESCVFIPCITGIA-GCSCKSK-VCYRN	6

^*^Number of identified sequences.

^**^Unidentified residues are underlined (Missing due to truncation or double digestion by butelase 2).

**Table 2:** Molecular diversity of cliotide represented by cT7.

Peptide derived from ctc7 gene	−10logP^a^	Mass	Error ppm	m/z	z	No. (C)	MOD^b^
GIPCGESCVFIPCTVTALLGCSCKDKVCYKN	148.48	3508.7559	−0.7	702.758	5	6	EA^c^
**G**IPCGESCVFIPCTVTALLGCSCKDKVCYKN^d^	67.06	3550.7664	−1	592.801	6	6	NT-Acetylation (+42.01); EA
GIPCGESCVFIPCTVTALLGCSCKDKVCY	39.27	3266.6179	−0.3	545.443	6	6	EA
GIPCGESCVFIPCTVTALLGCS	87.25	2341.1665	0.5	586.299	4	4	EA
GIPCGESCVFIPCTVTALLGC	89.92	2254.1345	−0.7	564.541	4	4	EA
GIPCGESCVFIPCTVTALLG	83.75	2108.0833	−0.5	528.028	4	3	EA
GIPCGESCVFIPCTVTALL	89.03	2051.0618	0.3	513.773	4	3	EA
GIPCGESCVFIPCTVTAL	77.69	1937.9777	−0.3	485.502	4	3	EA
**G**IPCGESCVFIPCTVTAL	37.86	1979.9883	−0.1	661.003	3	3	NT-Acetylation (+42.01); EA
GIPCGESCVFIPCTVTA	105.51	1824.8936	−0.1	457.231	4	3	EA
**G**IPCGESCVFIPCTVTA	47.35	1866.9042	−0.3	623.309	3	3	NT-Acetylation (+42.01); EA
GIPCGESCVFIPCTVT	72.32	1753.8564	−0.1	439.471	4	3	EA
GIPCGESCVFIPCTV	46.09	1652.8088	−0.4	551.943	3	3	EA
GIPCGESCVFIPCT	59.6	1553.7404	0	518.921	3	3	EA
GIPCGESCVFIPC	87.7	1452.6927	0.3	485.238	3	3	EA
GIPCGESCVFIP	45.56	1306.6414	0.3	436.555	3	2	EA
GIPCGESCVF	77.6	1096.5045	−0.2	366.509	3	2	EA
IPCGESCVFIPCTVTALLGCSCKDKVCYKN	34.93	3451.7344	5.8	576.3	6	6	EA
PCGESCVFIPCTVTA	42.08	1654.7881	−0.1	552.603	3	3	EA
PCGESCVFIPCTVT	40.16	1583.751	0.7	528.925	3	3	EA
IPCTVTALLGC**S**CKDKVCYKN	43.84	2592.3147	−0.4	519.47	5	4	EA; Hexose (S) (+162.05)
IPCTVTALLGCSCKDKVCYK	129.36	2430.262	0.2	487.06	5	4	EA
CTVTALLGCSCKDKVCYK	123.83	2220.125	−0.4	445.032	5	4	EA
TVTALLGCSCKDKVCYK**N**	56.02	2075.0576	5.5	519.775	4	3	EA; Deamidation (N) (+.98)
TVTALLGC**S**CKDKVCYKN	46.32	2236.1265	−0.2	560.039	4	3	EA; Hexose (S) (+162.05)
TVTALLGCSCKDKVCYKN	118.62	2074.0737	−0.5	519.526	4	3	EA
VTALLGCSCKDKVCYKN	97.33	1973.026	−0.1	494.264	4	3	EA
TALLGCSCKDKVCYKN	103.44	1873.9576	0.2	469.497	4	3	EA
ALLGCSCKDKVCYKN	39.47	1729.8677	0	433.474	4	3	EA
LLGCSCKDKVCYK**N**GIPCGESCVFIPCTVTA	74.15	3509.74	0.3	502.399	7	6	EA; Deamidation (N) (+.98)

^a^In Peaks PTM function, the p-value is converted from the linear discriminative function score. A higher −10logP value indicated a more confident sequencing result. −10lgP value > 25 is equivalent to false discovery rate (FDR) < 0.03%.

^b^MOD: side-chain modifications and chemical derivatization.

^c^All Cys residues were alkylated with ethylamine and labeled as EA.

^d^Residues with modifications are bold and underlined.
