# Supplementary material for: A high-throughput peptidomic strategy to decipher the molecular diversity of cyclic cysteine-rich peptides
Source: Sci Rep. 2016 Mar 11;6:23005. doi: 10.1038/srep23005 (PMC4786859; doi:10.1038/srep23005)
Supplement: Supplementary Information [file srep23005-s1.doc]

**Supplementary Information**

A high-throughput peptidomic strategy to decipher the molecular diversity of cyclic cysteine-rich peptides

Aida Serra,^+^ Xinya Hemu,^+^ Giang K T Nguyen, Ngan T K Nguyen, Siu Kwan Sze, James P Tam^*^

School of Biological Sciences, Nanyang Technological University, 60 Nanyang Drive, Singapore 637551

^+^These authors contributed equally to this work

^*^ Corresponding author: JPTam@ntu.edu.sg

**Supplementary Results**

| **A** | **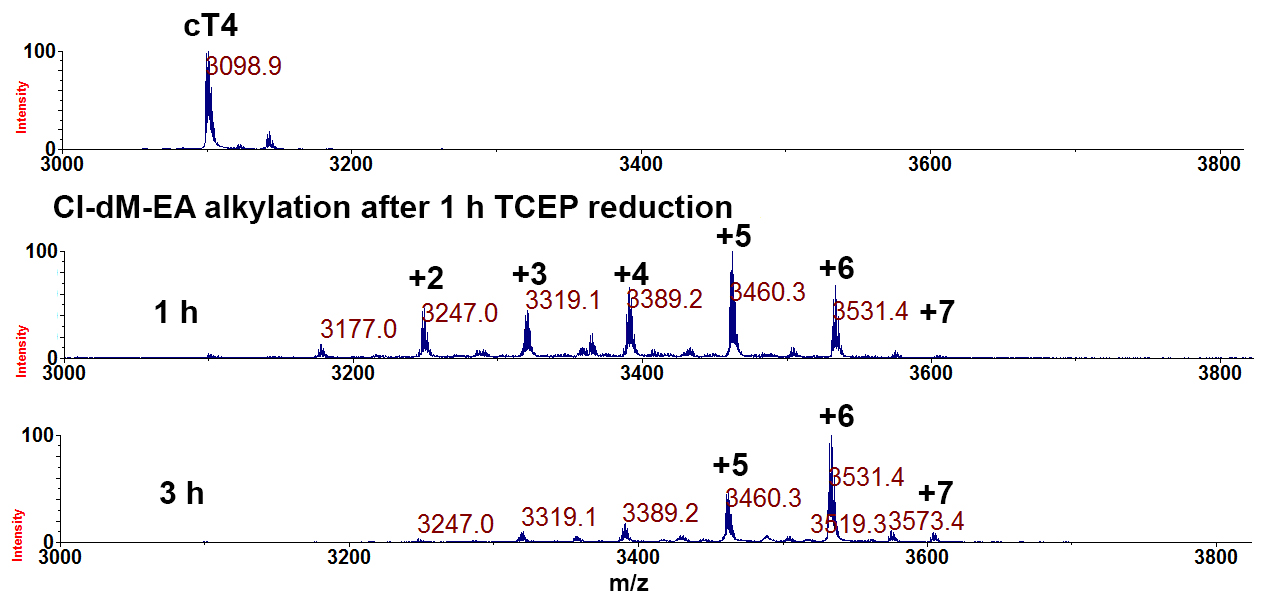** |
| --- | --- |
| **B** | **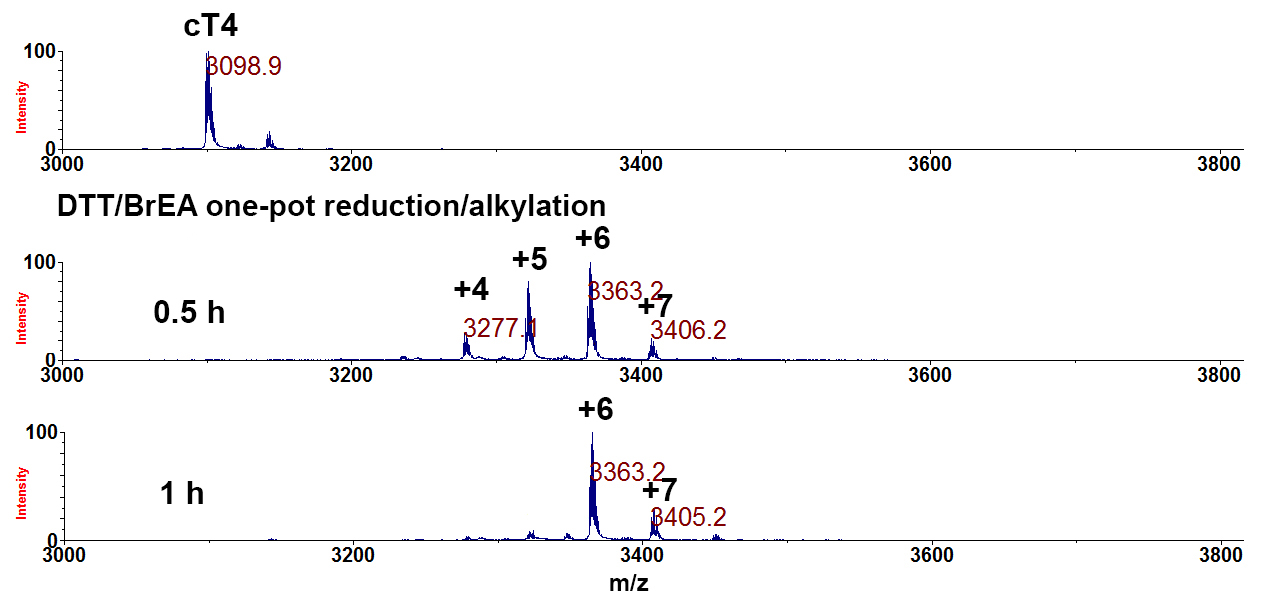** |

**Figure 1: Comparison of two-step and one-pot conditions for cyclotide reduction and alkylation of cT4.** (A) Two-step condition: (1) 0.1 mM cT4 was reduced with 50 mM TCEP at 37 °C for 1 h. (2) Alkylated with 2-chloro-*N,N*-dimethylethylamine (0.25 M) in 50 mM Tris-HCl (pH 7.8) and incubated at 25 °C for 3 h. (B) One-pot condition: 0.1 mM cT4 was mixed with 30 mM DTT and 60 mM BrEA in a Tris-HCl buffer (0.2 M, pH 8.6) and reaction was performed at 55 °C for 1 h.

| **A** | 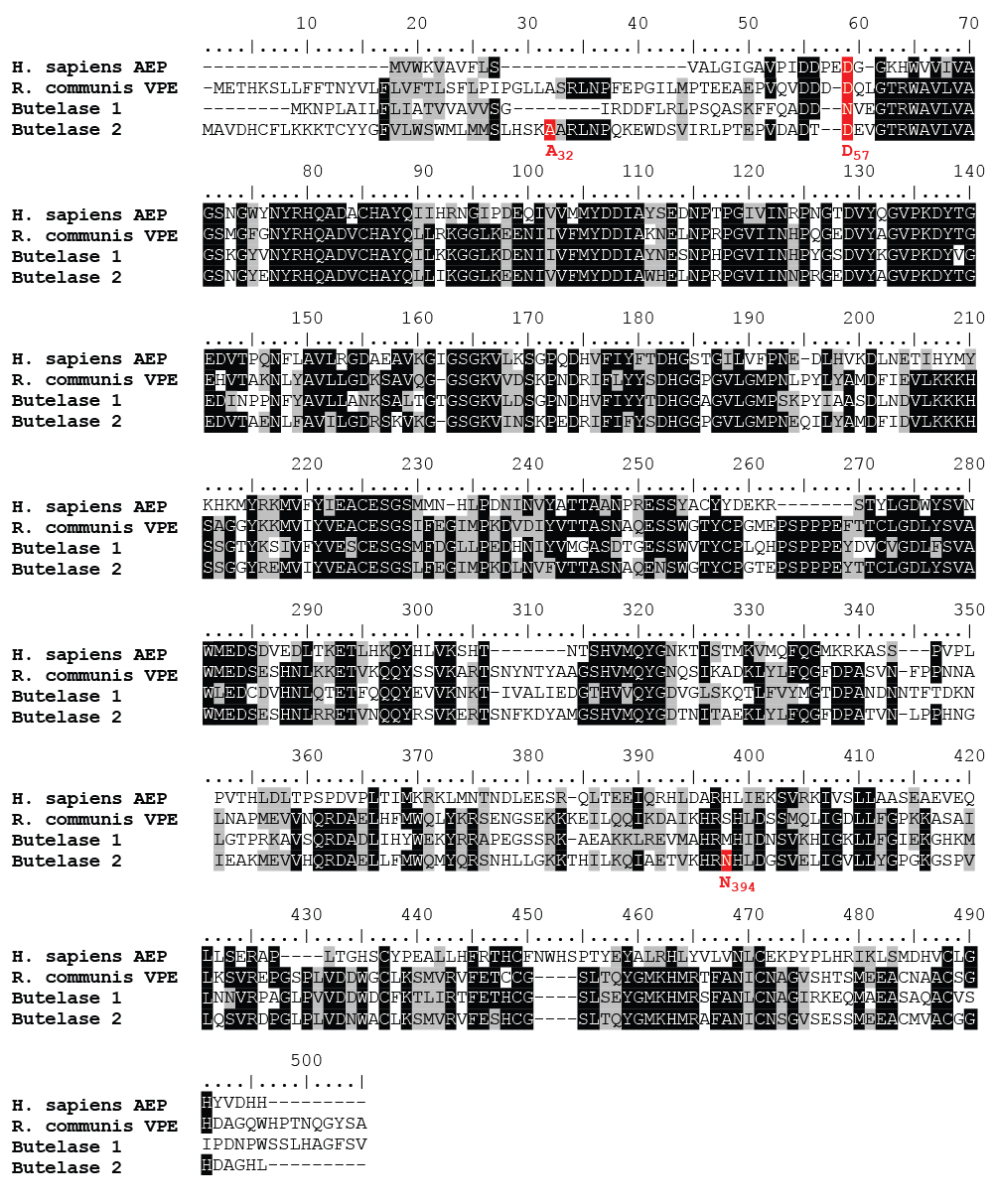 | | |
| --- | --- | --- | --- |
| **B** | **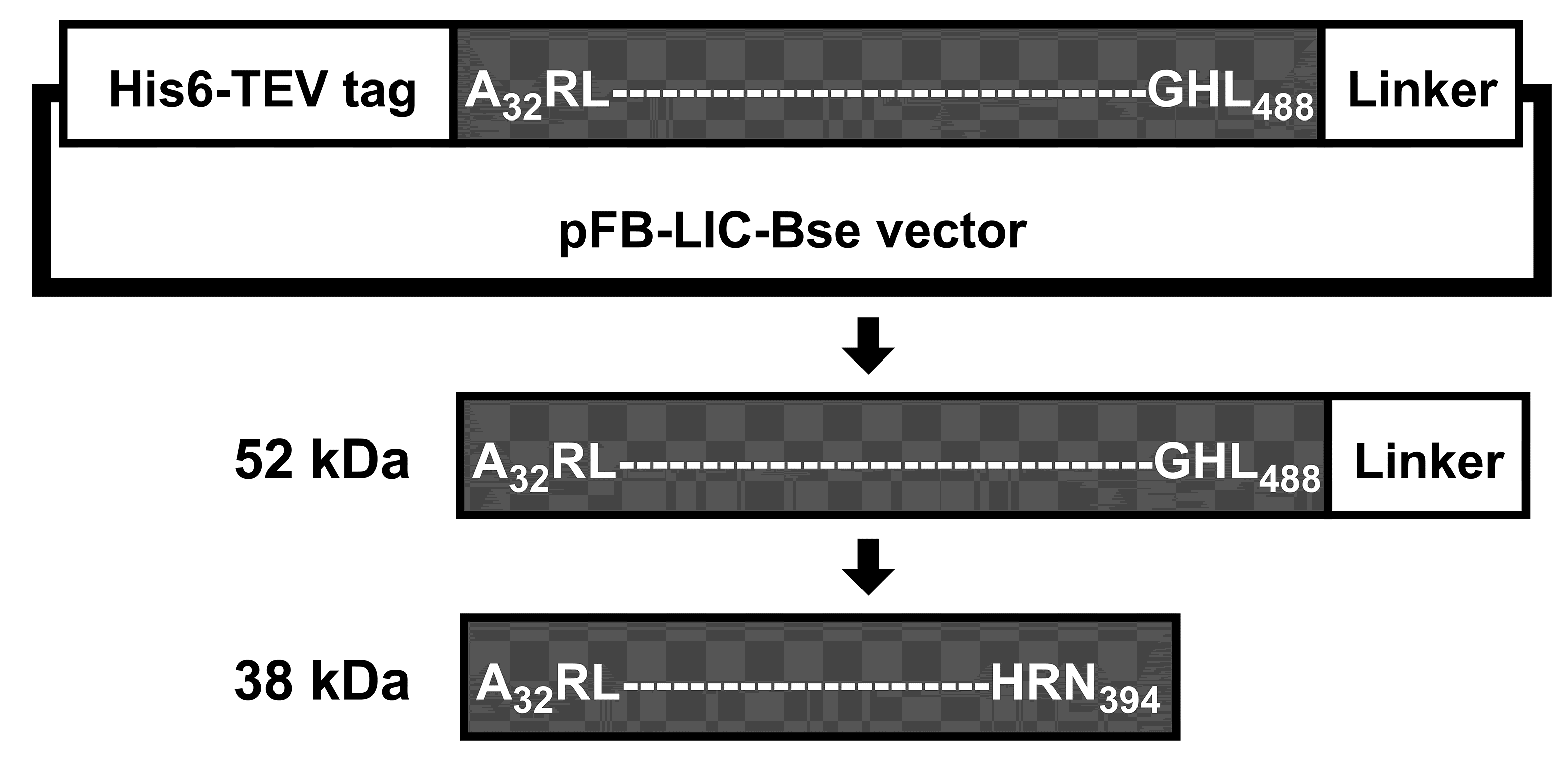** | **C** | **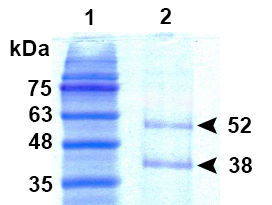** |

**Figure 2. Putative auto-activation sites of butelase 2.** (A) Autocatalytic sites in butelase 2 predicted from sequence alignment with mammalian and plant AEP. Protein sequence alignment was carried out using BioEdit. Identical amino acids are shaded in black and si­milar amino acids are shaded in grey. The signal peptide was predicted using SignalP server. The predicted autocatalytic sites of butelase 2 are boxed in red as D57 and N394. (B) The full-length precursor of butelase 2 without the signal sequence was cloned into the pFB-LIC-Bse vector for recombinant expression in Sf9 insect cells. The expressed protein after removing His6-TEV tag was 52 kDa and the active form after auto-activation at pH 5.0 was 38 kDa. (C) SDS-PAGE analysis of expressed butelase 2 after 2 h auto-activation. The mixture of partially activated butelase 2 was used for cliotide digestion without further purification. Lane 1, protein ladder. Lane 2, protein sample.

**Table S1**. Parameters for RNA assembly

| **Software** | **Version** | **Parameters** |
| --- | --- | --- |
| Trinity | Release-20121005 | --seqType fq --min_contig_length 100  --group_pairs_distance 250 --path_reinforcement_distance 85  --min_kmer_cov 2 |
| TGICL | V2.1 | -l 40 -c 10 -v 20 |
| Phrap | Release 23.0 | -repeat_stringency 0.95 -minmatch 35 -minscore 35 |


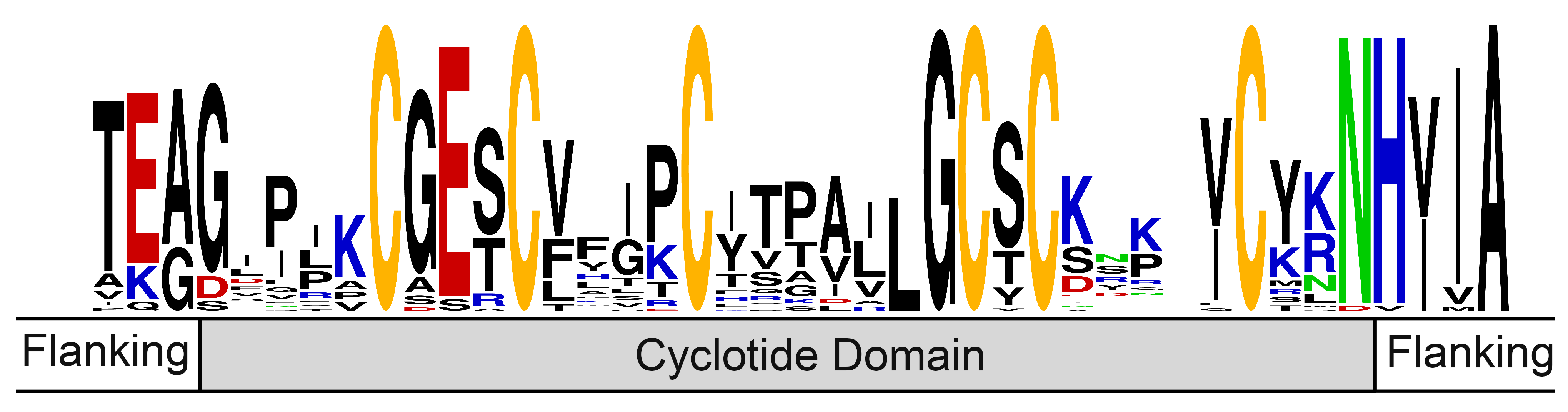


**Figure 3**. Sequence logo of cliotide precursors including N- and C-terminal flanking sequences. P1’ position after Asx was highly conserved as His/Val and P2’ position was highly conserved as hydrophobic Val/Ile.

**
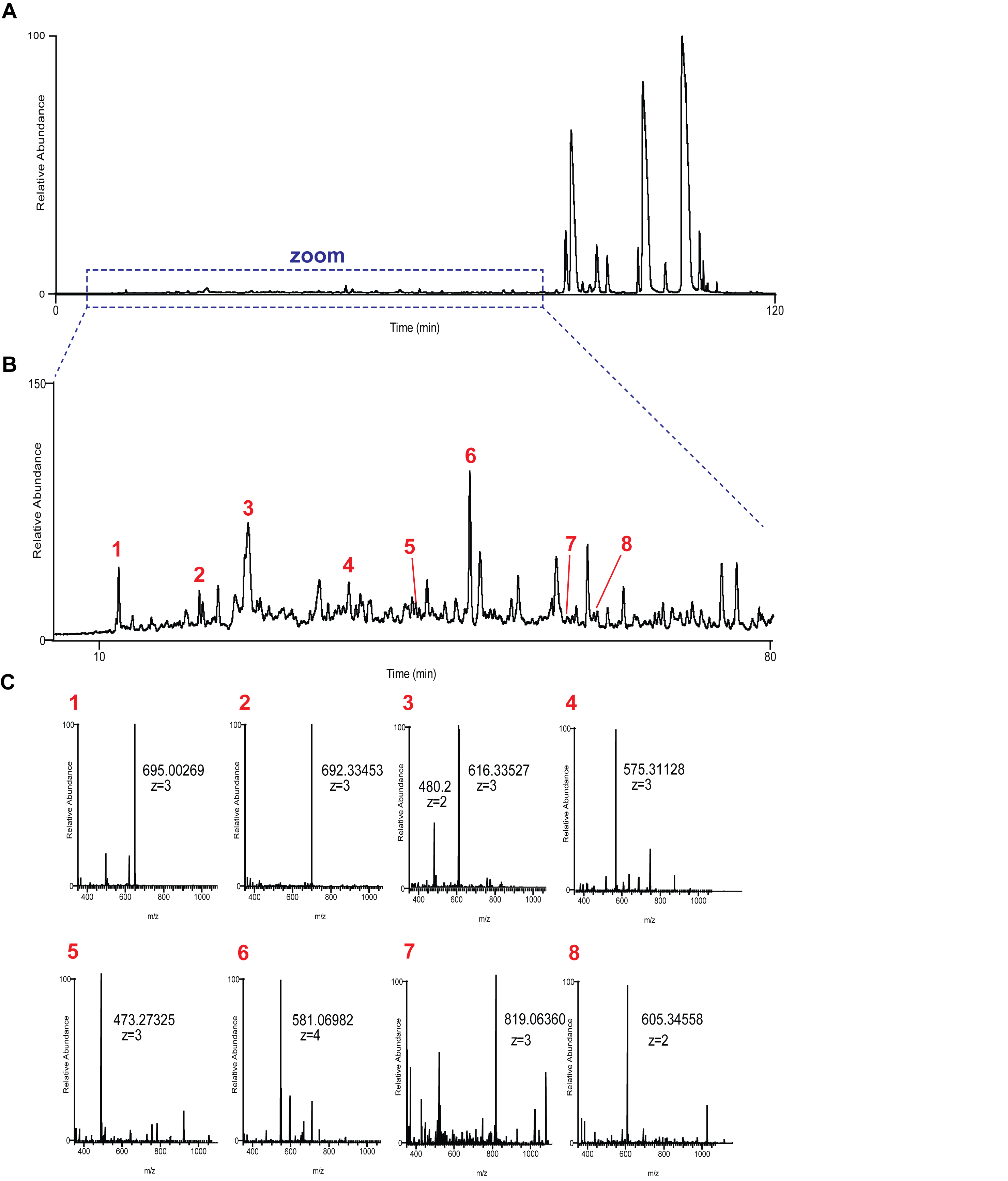
**

**Figure 4:** Mass spectrometry analysis of the non-treated raw extract of *C. ternatea* plant. (A) Base peak intensity chromatogram corresponding to fraction #2 of the non-treated raw extract obtained by SCX-LC. (B) Zoomed view of the low intensity peaks detected by LC-MS in fraction #2 of the non-treated raw extract. (C) Eight MS profiles corresponding to peptides with mass < 3000 Da randomly selected from the LC-MS profile. Peak localization in the LC-MS profile are identified by numbers (1-8).

**
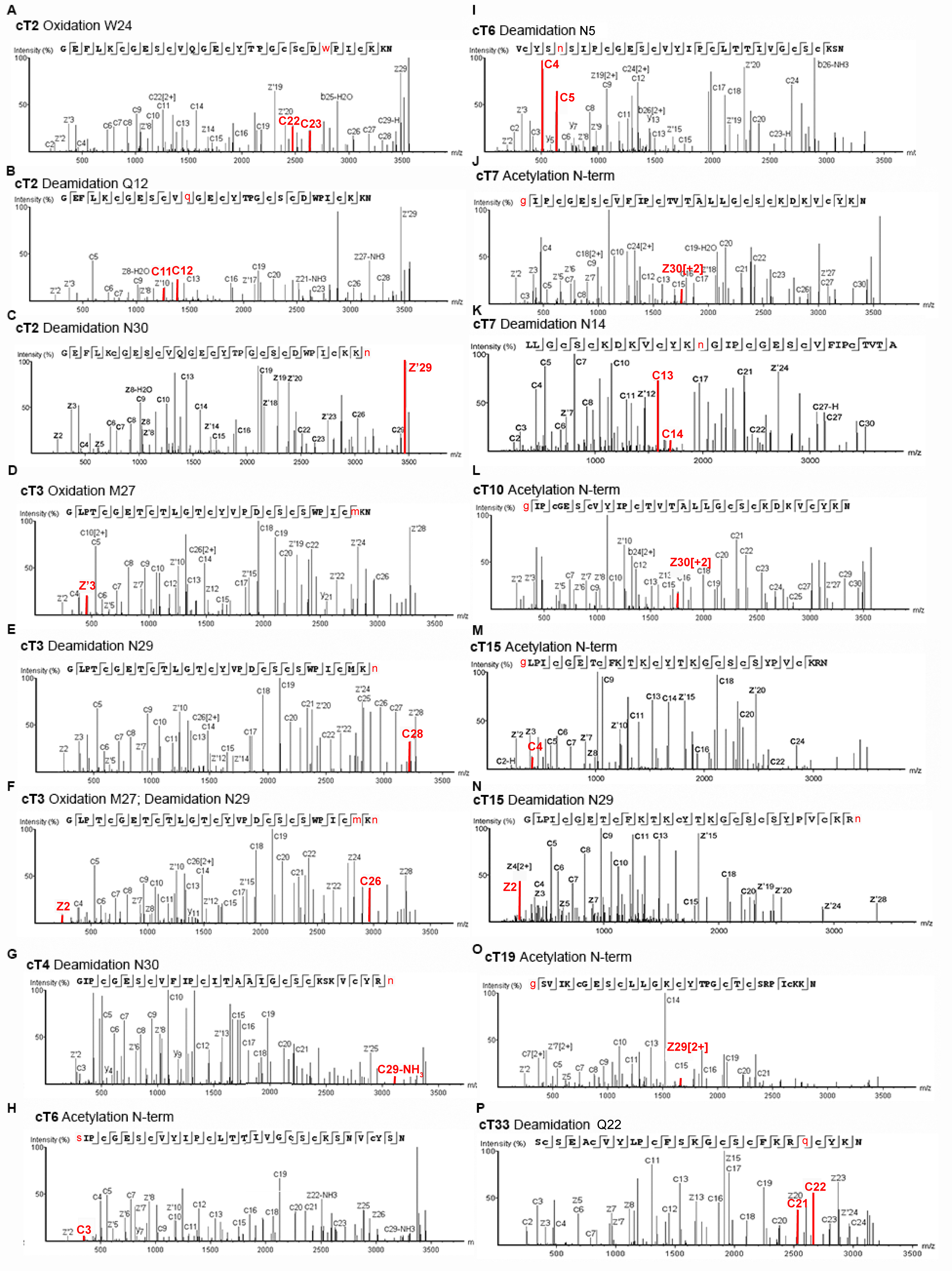
**

**Figure 5:** MS/MS spectra showing post-translational modifications on full sequences. Fragments are labeled with *c*-, *z*-, *z*+1 (*z*’), *z*+2 (*z*(+2)), *b*- and *y*- ions. Fragments involved in PTM identifications are highlighted in red.

**
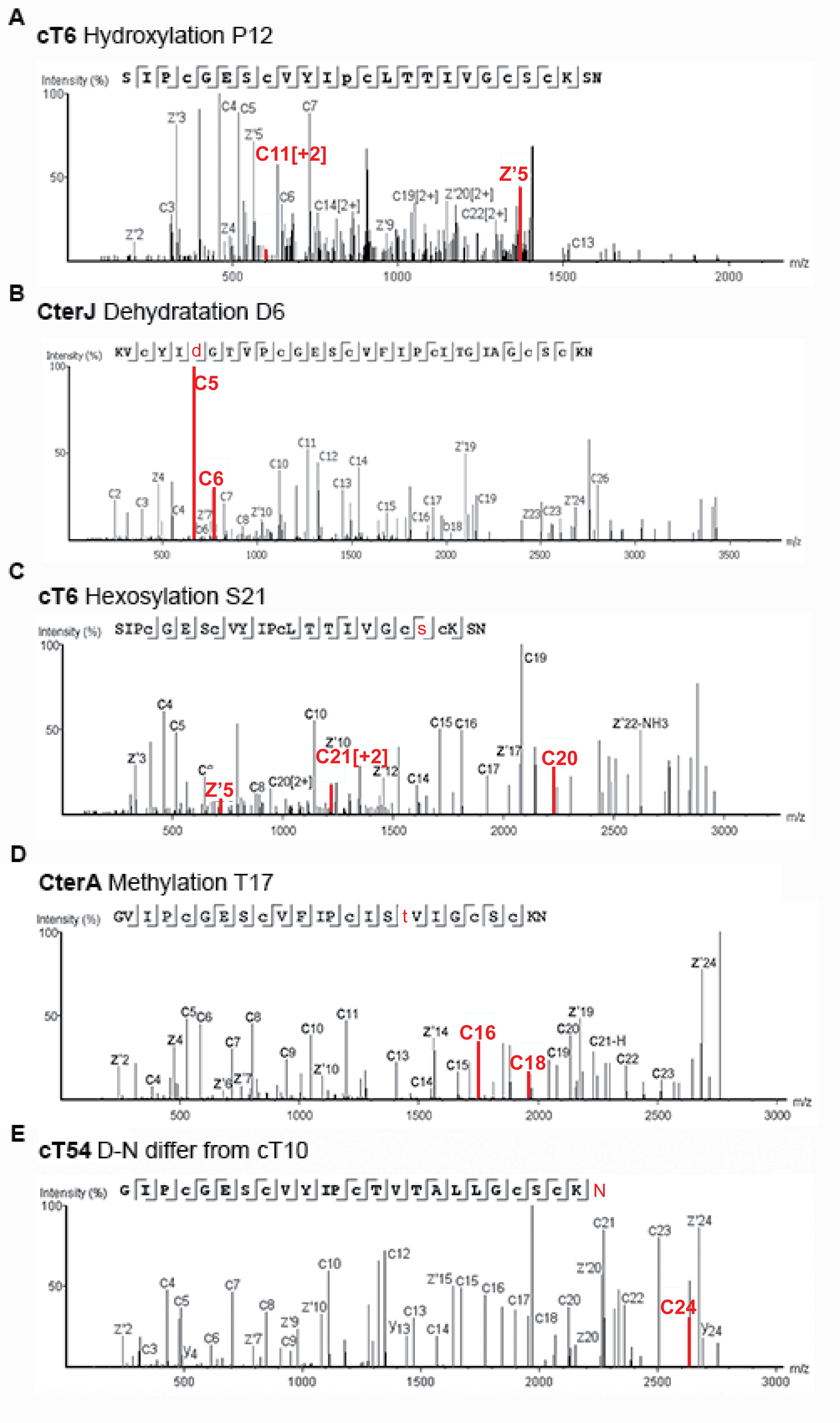

Figure 6:** MS/MS spectra showing post-translational modifications and the new cliotide cT54. Fragments are labeled with *c*-, *z*-, *z*+1 (*z*’), *z*+2 (*z*(+2)), *b*- and *y*- ions. Fragments involved in PTM identifications are highlighted in red.
